# Risk and Predisposing Factors for Suicide Attempts in Patients with Migraine and Status Migrainosus: A Nationwide Population-Based Study

**DOI:** 10.3390/jcm7090269

**Published:** 2018-09-11

**Authors:** Tomor Harnod, Cheng-Li Lin, Chia-Hung Kao

**Affiliations:** 1 Department of Neurosurgery, Hualien Tzu Chi General Hospital, Buddhist Tzu Chi Medical Foundation, Hualien 970, Taiwan; tomorha@yahoo.com.tw; 2 College of Medicine, Tzu Chi University, Hualien 970, Taiwan; 3 Management Office for Health Data, China Medical University Hospital, Taichung 404, Taiwan; orangechengli@gmail.com; 4 College of Medicine, China Medical University, Taichung 404, Taiwan; 5 Graduate Institute of Biomedical Sciences and School of Medicine, College of Medicine, China Medical University, Taichung 404, Taiwan; 6 Department of Nuclear Medicine and PET Center, China Medical University Hospital, Taichung 404, Taiwan; 7 Department of Bioinformatics and Medical Engineering, Asia University, Taichung 413, Taiwan

**Keywords:** cohort study, migraine, National Health Insurance, suicide

## Abstract

Objective: To investigate the risk and risk factors for suicide attempt by patients with regular migraines (RM) and status migrainosus (SM) in Taiwan. Methods: We analyzed a subset of the National Health Insurance Research Database of Taiwan and enrolled patients (≥20 years old) who had ever received a diagnosis of RM or SM between 2000 and 2012 in the RM and SM cohort. The SM cohort included 13,605 patients, the RM cohort had 21,485 patients, and the comparison cohort contained approximately four times that many patients. We calculated the adjusted hazard ratios and 95% confidence intervals (CI) for suicide attempts after adjusting for age, sex, monthly income, urbanization level, occupation, and comorbidities. Results: The SM cohort had a 1.81-fold risk of attempting suicide (95% CI = 1.14–2.89) compared to the comparison cohort. Other factors that predispose patients with SM to attempt suicide include the following: female sex, relatively young age (<50 years old), and low monthly income (<15,000 New Taiwan Dollars, approximately equivalent to 495 US Dollars). Additionally, the risk of attempting suicide only increased in patients who had been diagnosed with SM for longer than five years. Conclusion: SM is associated with a higher risk for suicide attempt in migraineurs in Taiwan. This finding is important to clinicians and government officials seeking to prevent patients from attempting suicide in Taiwan and other similar East Asian countries.

## 1. Introduction

A migraine is the most frequently diagnosed type of headache at neurological clinics across Asian developing countries, accounting for 66.6% of all headache services (range: 50.9–85.8%) [1]. Numerous epidemiological studies conducted in Western developed countries during the past two decades have found that migraines affect approximately 20% of the general population [2,3]. Globally, migraines are approximately twice as prevalent in females as in males, particularly affecting young and middle-aged females with menstrual cycles [3,4,5]. Migraines affect the central nervous system and impair daily life for those affected, cause comorbid psychological conditions, and increase the risk of cardiovascular diseases for patients [6,7]. Investigations continue regarding the detailed mechanism and risk factors for migraines.

Studies disagree about whether migraineurs experience an elevated risk of suicidal thoughts or actions [7,8]. We would not usually see a headache as a common risk factor for suicidal actions. However, we discovered that most of the previous studies of suicidal behaviors exhibited by the patients were investigated in patients with general or regular migraines (RM), and did not focus on patients with a treatment-resistant or status migrainosus (SM) condition. SM poses a unique and difficult challenge to headache specialists. Patients with SM have more disability than patients with a classic, general migraine and thus may require specific pharmacological and psychological strategies to reduce the burden of the disease [9,10]. Therefore, further investigation is required to compare the correlated risks of suicide attempts in patients with RM and SM to elucidate future treatment strategies among these specific ones.

Located in East Asia, Taiwanese people share the cultural mores of the Han people and have a heritage similar to the people in the developing societies of China and Southeast Asian countries [11]. Additionally, a recent study demonstrated that people from Taiwan, Hong Kong, and Japan exhibit similar patterns of suicidal behaviors that differ from those exhibited in Western countries [12]. If we identify risk factors and patterns of suicidal behavior in Taiwanese migraine patients, our findings may also apply to migraine suffers in Hong Kong, Japan, China, and Southeast Asian countries. The Taiwanese government has administered a healthcare system covering approximately 99.9% of the population for the past two decades [13,14]. We used a nationwide population-based database to investigate risk factors for suicide attempt by patients with SM. The findings of this study might constructively inform the development and implementation of a more effective suicide prevention system in clinics throughout Taiwan.

## 2. Methods

### 2.1. Data Source

We conducted this retrospective cohort study using the Longitudinal Health Insurance Database 2000 (LHID 2000) from the Taiwanese National Health Insurance (NHI) program that enrolls more than 23 million individuals, representing approximately 99.9% of the population of Taiwan [13,14]. The LHID 2000 and NHI program details have been described in previous studies [15,16]. The Research Ethics Committee of China Medical University and Hospital in Taiwan approved the study (CMUH104-REC2-115-CR3).

### 2.2. Participants

This study identified patients 20 years old or older with a diagnosis of RM (ICD-9-CM code 346 except 346.9) or SM (ICD-9-CM code 346.9, and without code 346.90 or 346.91) from 1 January 2000 to 31 December 2012 as the RM and SM cohort. We used the date of diagnosis of RM or SM for the index date, and excluded patients who had ever had suicide attempts and self-inflicted injuries (ICD-9-CM codes E950-E959) history before the index date. Patients younger than 20 years old or without age and sex data were also excluded. We randomly selected four times as many subjects without a migraine diagnosis as RM and SM cohorts from the entire LHID 2000 beneficiary roster to comprise each comparison cohort. We frequency matched the comparison subjects with the RM and SM patients by sex, age (five-year age groups), and year of index date. The comorbidities of schizoprenia (ICD-9-CM 295), depression (ICD-9-CM 296.2, 296.3, 296.82, 300.4, and 311), alcohol-related illness (ICD-9-CM 291, 303, 305.00, 305.01, 305.02, 305.03, 571.0, 571.1, 571.3, 790.3, and V11.3), anxiety (ICD-9-CM 300.00), mental disorders (ICD-9-CM 290–319), and insomnia (ICD-9-CM 307.4 and 780.5) were identified and adjusted in the analysis.

### 2.3. Outcome Measurements

The primary outcome of interest in this study was attempted suicide. To determine the incidence density rate for attempted suicide, we assessed each patient from the index date to the date a suicide attempt occurred, until the patient was removed from LHID 2000 because of death, or until the study concluded on 31 December 2013, whichever occurred first.

### 2.4. Statistical Analysis

We used chi-square testing to determine the differences in sociodemographic status and comorbidities between the SM and comparison cohorts, and used the Student *t*-test to examine the mean ages and mean follow-up periods between the two cohorts. We estimated the cumulative incidences of suicide attempts for both the SM and comparison cohorts using the Kaplan-Meier method, and examined the difference between the two curves using the log-rank test. We estimated the incidence densities of suicide attempts for different risk factors and stratified them by age, sex, monthly income, urbanization level, occupation category, comorbidity, and follow-up period. Univariable and multivariable Cox proportional hazards regression models were used to assess the hazard ratios (HR) and 95% confidence intervals (CI) of suicide attempts in different categories. The variables we analyzed in the multivariable model were age, sex, monthly income, and comorbidities of schizophrenia, depression, alcohol-related illness, anxiety, mental disorders, and insomnia, which were significantly different in the univariable Cox model. All of the above statistical methods were also used to compare the risk of attempted suicide between the RM and comparison cohorts. We used the scaled Schoenfeld residuals to test the proportional hazard model assumption. Because the proportional hazard model assumption was violated (*p* value = 0.003), we stratified the follow-up duration (≤5 years and >5 years) to address the violation of the proportional hazard model assumption. Additionally, we regarded the death event as a competing event to estimate subhazard ratios (SHRs) and 95% CI of suicide attempts using the Fine and Gray method [17]. We used SAS^®^ 9.4 software (Statistical Analysis System Institute Inc. (SAS Institute), Cary, NC, USA) for data analysis and considered a two-tailed *p* value <0.05 as indicative of a statistically significant result.

## 3. Results

**Figure 1:**
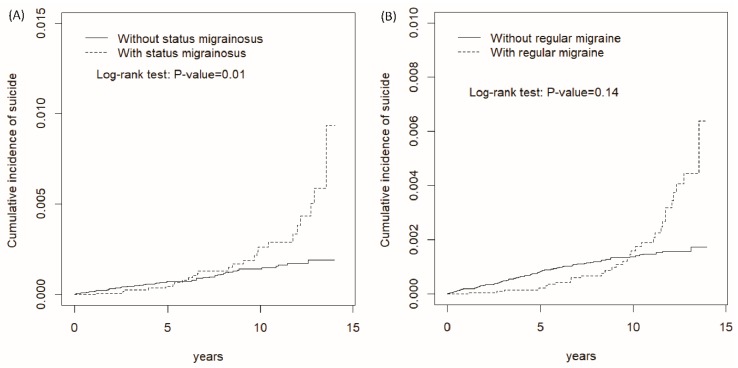
Cumulative incidences of suicide attempts for patients with status migrainosus and the comparison cohort (**A**), and for patients with regular migraines and the comparison cohort (**B**).

We enrolled 13,605 patients in the SM cohort and 54,379 subjects in the comparison cohort. There was no statistical significance for distribution of sex, age, or comorbidities between the SM and comparison cohorts. Similarly, 21,483 patients in the RM cohort were matched with 85,840 comparison subjects according to the frequency matched. In both cohorts, most of the subjects were female (74.2%) and younger than 50 years old (64.2%). The mean age was 45.7 ± 14.8 years old for the SM cohort and 45.6 ± 15.1 years old for the comparison cohort. Most subjects in the SM and comparison cohorts had income levels <15,000 New Taiwan Dollars (NTD, approximately equivalent to 495 US Dollars) per month (37.1% vs. 38.5%), and lived in highly urbanized areas (59.2% vs. 60.7% in urbanization levels 1 and 2). The primary comorbidity in the study cohorts was insomnia (68.9%), followed by anxiety (37.7%), depression (13.3%), and mental disorders (11.3%) (Table 1). Meanwhile, there were no significant differences noted for sex, age, and comorbidity statuses between the RM and the matched comparison cohorts. The average follow-up duration was 7.35 ± 3.78 years for the SM cohort and 7.26 ± 3.79 years for the comparison cohort. The Kaplan-Meier graph shows that the cumulative incidence of suicide attempts was higher for the SM cohort than the comparison cohort throughout the 12-year follow-up period (*p* = 0.01 for the log-rank test) (Figure 1A). We noted that the cumulative incidences of suicide attempts were not significantly different between the RM and comparison cohorts (Figure 1B, log-rank test: *p* = 0.14).

The overall incidence density of suicide attempt was 1.42 per 10,000 person-years in the comparison cohort and 2.60 per 10,000 person-years in the SM cohort. The corresponding adjusted HR (aHR) for suicide attempt was 1.81 (95% CI = 1.14–2.89) compared to comparisons, after adjusting for age, sex, monthly income, and the comorbidities of schizophrenia, depression, alcohol-related illness, anxiety, mental disorders, and insomnia. Compared to the 50–64 year age group, the group with subjects 49 years old or younger had a significant 3.27-fold risk for attempting suicide (95% CI = 1.56–6.87). Compared to males, females had an aHR of 2.67 (95% CI = 1.31–5.43) for attempting suicide. The group with a low monthly income (<15,000 NTD) had an aHR of 2.44 (95% CI = 1.24–4.78) for attempting suicide compared to those with a high monthly income (≥20,000 NTD). The risk for attempting suicide was higher in subjects with depression (aHR = 2.15, 95% CI = 1.27–3.65), alcohol-related illness (aHR = 4.32, 95% CI = 2.22–8.41), anxiety (aHR = 1.95, 95% CI = 1.15–3.31), and insomnia (aHR = 1.91, 95% CI = 1.06–3.46) (Table 2). No significant difference was observed in the overall risks of suicide attempts between the RM and comparison cohorts (Table 3).

After we adjusted for all the confounding factors and considered the competing risks of death, the SM cohort retained a significantly higher risk of attempting suicide than that of the comparison cohort (adjusted SHR = 1.99, 95% CI = 1.24–3.18) (Table 4). Notably, as presented in Table 5, the incidence rate of liquid or solid poisoning was 2.63-fold higher for those with SM than the comparison cohort (aHR = 2.63, 95% CI = 1.47−4.71).

## 4. Discussion

This study discovered that the cumulative incidence for suicide attempt was markedly increased in the SM cohort compared to the comparison cohort. Our results indicate that patients ever diagnosed with SM in Taiwan have a 1.81-fold risk of attempting suicide compared to the comparisons, and female patients were more likely than male ones to attempt suicide. The competing risk study demonstrates that SM is highly associated with the increased risk of suicide attempt. This study also identified some notable factors that dispose patients with SM to suicide attempts. These factors are an age younger than 50 years old, female sex, and a low monthly income of <15,000 NTD. Our findings diverge from recent reports of the suicidal tendencies in the general population of Taiwan, which found that male and elderly individuals predominantly engage in suicidal behaviors [12]. Additionally, we found that the risk of suicide attempts specifically increases in patients at five years following the diagnosis of SM, but not since the beginning of follow-up period. This implied that the severity and duration of chronic migraine would together play critical roles in a patient’s psychological burden and the consequence of suicide attempt.

In addition to the SM diagnosis, female sex, younger age (<50 years old), and low monthly income (<15,000 NTD) are socioeconomic factors affecting the patients. Long-term health-related support from society and government is usually required to improve life spans and quality of life for patients with chronic and treatment-resistant disorders such as SM. Previous studies reported that a low socioeconomic status and relatively non-supportive societies exert a strong negative influence on the life spans of patients with chronic disorders [18,19,20], with suicide being a relatively common cause of death for these patients [21]. Our study discovered a strong association of increased risk of attempted suicide in patients ever diagnosed with SM, and we hope our findings highlight the urgency of providing more proper and effective treatment to SM patients in the future.

Ethnic and cultural differences between Asian and Western societies may account for the difference between our results and those of similar studies conducted in Western countries [7,8]. Ethnic and cultural heritage is strongly associated with cognition, interpretation, judgment of personal values and dignity, and response to painful stimulation [22,23,24]. Some studies have suggested that perception sensitivity and tolerance to pain differ among various ethnic groups, finding that interdependent individuals from Asian societies (e.g., females or young adults in Taiwan or China) tend to suppress emotional suffering more [24,25]. Consequently, as an intolerable pain condition based on chronic migraines, SM alters the neuroendocrinal, neurophysiological, and neuropsychological conditions of patients, and interacts with sociocultural factors to impel patients to attempt suicide to end their suffering.

Our study informs documents that self-poisoning is the most common method of suicide attempts in these patients, and preventive precautions by caregivers or family should account for this. Further, the risk of suicide attempt was higher in patients with SM and several psychiatric comorbidities, such as depression, alcohol-related illness, anxiety, and insomnia, but not in those with schizophrenia or mental disorders. A strong coexisting condition between migraines and depression has usually been observed [9,10]. There is an independent relationship between migraines and depression, and depression and suicide. However, the rate of diagnosed depression was only 13.3% in the SM cohort in this study, which implied that depression is currently under-diagnosed in patients with migraines. Furthermore, due to the fact that we controlled for most psychiatric comorbidities in the analysis and the study investigated a nationwide, representative, population-based sample with little risk of recall and selection bias, our findings should be considered convincing and should warrant the development of a more efficient suicide prevention system in Taiwan.

This study nevertheless has some limitations. First, we could not directly contact patients because their identities were anonymized in the data set. Therefore, the study design did not include details about the headaches of the RM and SM patients, such as duration and frequency of headaches, the psychological burden caused by headaches, whether they occurred with any other particular kind of pain or illness, and whether or how the headaches were treated with medication; thus, none of these could be analyzed in relation to suicide attempts in this study. Whether any medications for preventing or treating migraines might be associated with suicidal ideation or suicide attempts remains unknown and should be the object of further study. We also could not determine the coping skills of the patients in this study, whether and to what extent they received support from government or society, or whether they experienced suicidal ideation before attempting suicides not recorded in the database. Second, our data set only included patients with RM and SM and suicide attempts treated at inpatient facilities. Any rare cases of suicide occurring outside a hospital or cases of suicide attempts without subsequent admission to a hospital fell outside the scope of our study. The NHI program has been conducted for over 20 years, covering more than 99% of Taiwan’s population and it guarantees the residents of Taiwan equal access to medical services, regardless of their socioeconomic status, background, and whether critical problems exist. Taiwan’s universal healthcare system displays very few disparities in accessing inpatient services and ultimate outcomes between different hospitals and areas in Taiwan [26,27]. We intentionally designed our study in this manner to achieve results with high validity, even though this might have introduced a slight possibility of underestimation bias into our results. Third, although the National Health Insurance program performs quarterly expert reviews to ensure the accuracy of claims filed and false claims are heavily sanctioned, a few degrees of miscoding and underdiagnoses of suicide events may nevertheless be present in the National Health Insurance Research Database. Our statistical results did, however, indicate that the sample size was sufficient to demonstrate a statistically significant relationship between SM and suicide attempts in migraineurs in Taiwan.

## 5. Conclusions

Suicide prevention is an important public health issue in Taiwan and globally. This study demonstrates that SM could be considered as a predisposing factor for patients with migraines to attempt suicide. Our findings provide important information for clinicians and government officials working to prevent suicide among patients with migraines in Taiwan and other similar East Asian countries.

## Figures and Tables

**Table 1:** Comparison of demographic characteristics and comorbidities in subjects with or without regular migraines and with or without status migrainosus.

	Status Migrainosus		*p*-Value	Regular Migraine		*p*-Value
	No	Yes		No	Yes	
	(*N* = 54,379)	(*N* = 13,605)		(*N* = 85,840)	(*N* = 21,483)	
Sex			0.95			0.95
Female	40,368(74.2)	10,096(74.2)		63,386(73.8)	15,859(73.8)	
Male	14,011(25.8)	3509(25.8)		22,454(26.2)	5624(26.2)	
Age stratified (years)			0.99			0.99
≤49	34,928(64.2)	8740(64.2)		56,524(65.9)	14,145(65.8)	
50–64	12,843(23.6)	3211(23.6)		19,440(22.7)	4864(22.6)	
65+	6608(12.2)	1654(12.2)		9876(11.5)	2474(11.5)	
Age, mean ± SD ^a^	45.6(15.1)	45.7(14.8)	0.42	44.9(15.2)	45.1(14.8)	0.23
Monthly income (NTD) ^†^			0.01			0.001
<15,000	20,922(38.5)	5043(37.1)		33,192(38.7)	7959(37.1)	
15,000–19,999	16,626(30.6)	4211(31.0)		25,934(30.2)	6687(31.1)	
≥20,000	16,831(31.0)	4351(32.0)		26,714(31.1)	6837(31.8)	
Urbanization level ^‡^			<0.001			0.001
1 (highest)	16,639(30.6)	3797(27.9)		26,485(30.9)	6222(29.0)	
2	16,363(30.1)	4251(31.3)		25,727(30.0)	6642(30.9)	
3	9296(17.1)	2249(16.5)		14,692(17.1)	3486(16.2)	
4 (lowest)	12,081(22.2)	3308(24.3)		18,936(22.1)	5133(23.9)	
Occupation category ^&^			0.001			0.001
Office worker	28,863(53.1)	7041(51.8)		45,943(53.5)	11,283(52.5)	
Laborer	20,873(38.4)	5464(40.2)		32,510(37.9)	8432(39.3)	
Other	4643(8.54)	1100(8.09)		7387(8.61)	1768(8.23)	
Comorbidity						
Schizophrenia	511(0.94)	137(1.01)	0.47	675(0.79)	184(0.86)	0.30
Depression	7214(13.3)	1810(13.3)	0.91	9918(11.6)	2494(11.6)	0.82
Alcohol-related illness	2045(3.76)	519(3.81)	0.77	2881(3.36)	734(3.42)	0.66
Anxiety	20,476(37.7)	5127(37.7)	0.95	30,253(35.2)	7578(35.3)	0.93
Mental disorders	6140(11.3)	1535(11.3)	0.98	9304(10.8)	2326(10.8)	0.96
Insomnia	37,477(68.9)	9379(68.9)	0.96	56,314(65.6)	14,097(65.6)	0.97

Chi-Square Test; ^a^ *t* test; ^†^ NTD, New Taiwan Dollar; ^‡^ urbanization level was determined by dividing residential areas into four levels on the basis of population density, where level 1 is the most urbanized and level 4 is the least urbanized; ^&^ other occupation categories included retired persons, unemployed persons, and low-income persons.

**Table 2:** Incidences and risk factors for suicide attempt with or without status migrainosus.

Variable	Event	PY	Rate ^#^	Crude HR (95% CI)	Adjusted HR ^$^ (95% CI)
Status migrainosus					
No	56	394,603	1.42	1.00	1.00
Yes	26	99,946	2.60	1.82(1.15, 2.90) *	1.81(1.14, 2.89) *
Age group (years)					
≤49	68	322,343	2.11	3.10(1.49, 6.45) **	3.27(1.56, 6.87) **
50–64	8	117,777	0.68	1.00	1.00
65+	6	54,429	1.10	1.68(0.58, 4.84)	1.58(0.55, 4.59)
Sex					
Female	73	369,296	1.98	2.74(1.37, 5.47) **	2.67(1.31, 5.43) **
Male	9	125,253	0.72	1.00	1.00
Monthly income (NTD) ^†^					
<15,000	40	171,388	2.33	3.14(1.68, 5.87) ***	2.44(1.24, 4.78) **
15,000−19,999	29	157,796	1.84	2.36(1.23, 4.54) *	1.89(0.98, 3.66)
≥20,000	13	165,366	0.79	1.00	1.00
Urbanization level ^‡^					
1 (highest)	26	148,541	1.75	1.46(0.71, 3.03)	
2	28	149,328	1.88	1.57(0.77, 3.24)	
3	10	83,682	1.20	1.00	
4 (lowest)	18	112,999	1.59	1.33(0.61, 2.87)	
Occupation category ^&^					
Office worker	33	259,306	1.27	1.00	1.00
Laborer	35	194,913	1.80	1.40(0.87, 2.25)	1.40(0.86, 2.29)
Other	14	40,330	3.47	2.76(1.48, 5.15) **	1.88(0.95, 3.69)
Comorbidity					
Schizophrenia					
No	79	489,986	1.61	1.00	1.00
Yes	3	4563	6.57	4.14(1.31, 13.1) *	1.76(0.54, 5.73)
Depression					
No	54	433,365	1.25	1.00	1.00
Yes	28	61,184	4.58	3.85(2.44, 6.08) ***	2.15(1.27, 3.65) **
Alcohol-related illness					
No	71	480,513	1.48	1.00	1.00
Yes	11	14,036	7.84	5.90(3.11, 11.2) ***	4.32(2.22, 8.41) ***
Anxiety					
No	30	307,897	0.97	1.00	1.00
Yes	52	186,652	2.79	2.93(1.87, 4.59) ***	1.95(1.15, 3.31) *
Mental disorders					
No	79	441,360	1.79	1.00	1.00
Yes	3	53,189	0.56	0.32(0.10, 1.01)	
Insomnia					
No	15	170,676	0.88	1.00	1.00
Yes	67	323,873	2.07	2.63(1.50, 4.63) ***	1.91(1.06, 3.46) *

CI, confidence interval; HR, hazard ratio; PY, person-years; ^#^ incidence rate per 10,000 person-years; ^$^ multivariable analysis included age, sex, monthly income, and comorbidity of schizophrenia, depression, alcohol-related illness, anxiety, mental disorders, and insomnia; ^†^ NTD, New Taiwan Dollar; ^‡^ urbanization level was determined by dividing residential areas into four levels on the basis of population density, where level 1 is the most urbanized and level 4 is the least urbanized; ^&^ other occupation categories included retired persons, unemployed persons, and low-income persons; * *p* < 0.05, ** *p* < 0.01, *** *p* < 0.001.

**Table 3:** Incidences and risk factors for suicide attempt with or without regular migraines.

Variable	Event	PY	Rate ^#^	Crude HR (95% CI)	Adjusted HR ^$^ (95% CI)
Regular migraine					
No	93	659,110	1.41	1.00	1.00
Yes	32	167,185	1.91	1.35(0.91, 2.02)	1.34(0.90, 2.00)
Age group (years)					
≤49	105	557,763	1.88	2.20(1.02, 4.72) *	2.77(1.28, 6.02) *
50–64	13	185,091	0.70	0.82(0.33, 2.06)	0.91(0.36, 2.30)
65+	7	83,441	0.84	1.00	1.00
Sex					
Female	106	616,795	1.72	1.88(1.16, 3.07) *	1.77(1.07, 2.93) *
Male	19	209,501	0.91	1.00	1.00
Monthly income (NTD) ^†^					
<15,000	58	290,561	2.00	2.69(1.63, 4.44) ***	1.92(1.11, 3.30) *
15,000−19,999	46	260,277	1.77	2.33(1.39, 3.90) **	1.88(1.12, 3.18) *
≥20,000	21	275,457	0.76	1.00	1.00
Urbanization level ^‡^					
1 (highest)	30	251,779	1.19	1.00	1.00
2	48	248,120	1.93	1.62(1.03, 2.56) *	1.47(0.93, 2.32)
3	17	138,799	1.22	1.03(0.57, 1.86)	0.96(0.53, 1.75)
4 (lowest)	30	187,598	1.60	1.34(0.81, 2.22)	1.26(0.75, 2.10)
Occupation category ^&^					
Office worker	52	439,466	1.18	1.00	1.00
Laborer	50	319,046	1.57	1.32(0.90, 1.95)	1.18(0.79, 1.78)
Other	23	67,784	3.39	2.89(1.77, 4.72) ***	2.19(1.27, 3.77) **
Comorbidity					
Schizophrenia					
No	118	820,075	1.44	1.00	1.00
Yes	7	6221	11.3	7.86(3.67, 16.9) ***	3.28(1.49, 7.223) **
Depression					
No	84	739,827	1.14	1.00	1.00
Yes	41	86,469	4.74	4.30(2.96, 6.25) ***	2.33(1.51, 3.60) ***
Alcohol-related illness					
No	114	805,688	1.41	1.00	1.00
Yes	11	20,607	5.34	4.00(2.15, 7.45) ***	2.61(1.37, 4.96) **
Anxiety					
No	49	539,650	0.91	1.00	1.00
Yes	76	286,645	2.65	2.96(2.07, 4.25) ***	1.87(1.23, 2.86) **
Mental disorders					
No	117	741,867	1.58	1.00	1.00
Yes	8	84,428	0.95	0.61(0.30, 1.24)	
Insomnia					
No	21	314,784	0.67	1.00	1.00
Yes	104	511,512	2.03	3.26(2.03, 5.22) ***	2.44(1.49, 4.00) ***

CI, confidence interval; HR, hazard ratio; PY, person-years; ^#^ incidence rate per 10,000 person-years; ^$^ multivariable analysis included age, sex, monthly income, urbanization level, occupation category, and comorbidity of schizophrenia, depression, alcohol-related illness, anxiety, and insomnia; ^†^ NTD, New Taiwan Dollar; ^‡^ urbanization level was determined by dividing residential areas into four levels on the basis of population density, where level 1 is the most urbanized and level 4 is the least urbanized; ^&^ other occupation categories included retired persons, unemployed persons, and low-income persons; * *p* < 0.05, ** *p* < 0.01, *** *p* < 0.001.

**Table 4:** Status migranosus cohort compared to comparison cohort subhazard ratio (SHR) of suicide attempts estimated using the competing-risks regression models.

	Competing-Risks Regression Models	
	Status Migrainosus	
	No	Yes
Status migrainosus		
Crude SHR (95% CI)	1(Reference)	2.90(1.77, 4.74) ***
Adjusted SHR ^†^ (95% CI)	1(Reference)	1.99(1.24, 3.18) **

Crude SHR, relative subhazard ratio; Adjusted SHR ^†^ multivariable analysis including all statistically significant risk factors in the univariable Cox model. ** *p* < 0.01, *** *p* < 0.001.

**Table 5:** Incidences and hazard ratios of different methods of suicide attempts between cohorts of status migrainosus and comparison.

Outcome	Status Migrainosus				Crude HR (95% CI)	Adjusted HR ^$^ (95% CI)
	No		Yes			
	Event	Rate ^#^	Event	Rate ^#^		
Liquid or solid poisoning (ICD-9-CM code E950)	28	0.71	19	1.90	2.66(1.49, 4.76) **	2.63(1.47, 4.71) **
Charcoal burning and poisoning by gases (ICD-9-CM code E952)	4	0.10	1	0.10	0.98(0.11, 8.77)	0.96(0.11, 6.60)
Hanging (ICD-9-CM code E953)	1	0.03	2	0.20	7.79(0.71, 85.9)	8.09(0.73, 89.3)
Cutting/piercing (ICD-9-CM code E956)	8	0.20	1	0.10	0.50(0.06, 3.96)	0.55(0.07, 4.43)
Jumping from high places (ICD-9-CM code E957)	2	0.05	0	0.00	-	-
Others (ICD-9-CM codes E951, E954, E955, E958, and E959)	13	0.33	3	0.30	0.91(0.26, 3.19)	0.93(0.27, 3.26)

CI, confidence interval; HR, hazard ratio; PY, person-years; ^#^ incidence rate per 10,000 person-years; ^$^ multivariable analysis included age, monthly income, and comorbidity of schizophrenia, depression, alcohol-related illness, anxiety, mental disorders, and insomnia. ** *p* < 0.01.

## Abbreviations

aHR	adjusted hazard ratio
CI	confidence interval
NHIRD	National Health Insurance Research Database
ICD-9-CM	International Classification of Diseases, Ninth Revision, Clinical Modification
